# Determining Adsorption Parameters of Potentially Contaminant-Releasing Materials Using Batch Tests with Differing Liquid-Solid Ratios

**DOI:** 10.3390/ma14102534

**Published:** 2021-05-13

**Authors:** Hirofumi Sakanakura, Kenichi Ito, Jiajie Tang, Mikako Nakagawa, Hiroyuki Ishimori

**Affiliations:** 1 Material Cycles Division, National Institute for Environmental Studies, 16-2 Onogawa, Tsukuba City 305-8506, Japan; mikakonakagawa55@gmail.com (M.N.); ishimori.hiroyuki@nies.go.jp (H.I.); 2 Center for International Relations, University of Miyazaki, 1-1, Gakuen Kibanadai-nishi, Miyazaki City 889-2192, Japan; itoken@cc.miyazaki-u.ac.jp; 3 Graduate School of Global Environmental Studies, Kyoto University, Yoshida-honmachi, Sakyo ku, Kyoto City 606-8501, Japan; tangjiajie0422@gmail.com

**Keywords:** batch leaching test, liquid-solid ratio, column percolation test, advection-dispersion model, adsorption–desorption equilibrium

## Abstract

Adsorption parameters such as the distribution coefficient are required to predict the release behavior of contaminants using advection-dispersion models. However, for potentially contaminant-releasing materials (PCMs) such as dredged sludge and coal ash, these parameters cannot be obtained by conventional adsorption tests. This study developed a method to determine adsorption parameters for PCMs from a set of batch tests conducted in parallel as a function of the liquid-solid ratio (LS-parallel test). This LS-parallel test was performed on sandy soil derived from marine sediment using liquid-solid ratios from 1 to 300 L/kg. The water-contact time was also changed from 10 min to 28 d to elucidate the kinetics or equilibrium of contaminants released from the sample. Adsorption parameters were successfully obtained if the substance was under adsorption control. A column percolation test was performed to confirm the effectiveness of the obtained parameters. Good agreements were observed for SO_4_^2−^ and B, but discrepancies remained for other substances such as F^−^ and As suggesting that improvements are necessary in both the LS-parallel test procedure and the advection-dispersion model.

## 1. Introduction

Solid materials such as dredged sludge, construction and demolition waste, steel slag, coal ash, and municipal solid waste incineration ash are anticipated to be recycled in construction works. In particular, these materials could be used to make features such as roadbeds, embankments, and landfill. However, these materials are also known to contain trace contaminants that might be released into the environment during their application. Therefore, the environmental impact of such potentially contaminant-releasing materials (PCMs) should be evaluated. Leaching is one of the most environmental important aspects, as contaminants might be transferred to downstream environments by contacting water, resulting in soil and groundwater pollution. Leaching tests can be used to evaluate this aspect of PCMs; the simplest types of leaching test are called single batch tests, which have been standardized by many organizations and countries (e.g., [1,2]). However, the test conditions are quite different from the real environment in that, for example, a single batch test does not have a water flow. The column percolation test (e.g., [3]) is closer to reality because it features water flowing through a column filled with a PCM sample. However, this test is more complicated and time-consuming than single batch testing. Therefore, efforts have been made to relate the results between batch and column percolation tests [4,5,6,7]. For example, comparable values were obtained between the amount eluted under a liquid-solid ratio (*LS_batch_*) of 10 L/kg in a batch test and the total amount released until a cumulative liquid-solid ratio (*LS_cum_*) of 10 L/kg was reached in a column percolation test (e.g., Reference [7]). However, when considering the realistic nature of results, column tests are also limited because their flow path length (e.g., 30 ± 5 cm [3]) is usually shorter than actual cases—in the case of an embankment the flow path length might reach several meters. Furthermore, the water-contact times of both batch tests (less than 24 h for most standards) and column tests (around or less than a few months for most standards) are shorter than the actual periods of application, which can sometimes last for decades.

Numerical model calculations can complement such discrepancies of leaching tests, for which the role of the leaching test is to provide the model parameters [8]. To this end, Reference [9] reviewed numerical models of the release of contaminants from PCMs. The single-mode first-order decay model [10,11,12] is the simplest model in which the outlet eluate concentration decreases exponentially. Non-precipitating and non-adsorbing substances can be applied to this model, assuming that the total amount of a substance is dissolved in a complete mixing box simulating pore water, and that it is gradually diluted and flows out along with freshwater inflow. However, the assumption that the entire system remains limited to one complete mixing box is not realistic when the flow path length increases. Thus, Reference [13] developed the continuously stirred tank reactor cascade model, based on double porosity. Similarly, Reference [14] developed the dual-mode first-order decay model that also covers non-precipitating and non-adsorbing substances, and can depict various decrease curve shapes. However, this model cannot scale up and extrapolate with consistent logic. The coupled chemistry transport model [15,16] considers solubility equilibrium with redox reactions and mass transfer by advection and dispersion. Reproducibility in blind simulations will be high if the model captures every chemical reaction appropriately. However, the reaction formula and the substances involved must be identified precisely for each PCM and for each application. Furthermore, the solubility equilibrium only occurs in very limited circumstances; for many PCMs such as dredged sludge, construction demolition waste, steel slag, and coal ash, the eluate concentration is often much lower than the solubility. In such cases, these substances are regarded as having precipitation-free and adsorptive characteristics.

A further study has showed the validity of the advection-dispersion model for describing the release behavior of contaminants from PCMs [17]. This model has been widely applied to capture the underground spreading of pollution [18]. The model assumes adsorption equilibrium, and can calculate the real scales and times of PCMs’ applications logically. Currently, it is necessary to perform a column percolation test to obtain the model parameters through the fitting of contaminant-release curves. If these parameters could be obtained from a simpler batch test method, then combined with model calculations, this could be quite practical for estimating the longer-term behavior of contaminants from PCMs in large scale scenarios. However, conventional adsorption tests (e.g., [19]) cannot provide adsorption parameters because the substance in question is also be eluted from the PCM itself.

In this study, a procedure was developed to obtain the adsorption parameters of PCMs through a set of batch tests with different liquid-solid ratios (LS-parallel test). This LS-parallel test was performed on a sandy soil derived from marine sediment, using not only different liquid-solid ratios, but also different water-contact times. This permitted discussion of the release mechanism regarding adsorption-control. Furthermore, a column percolation test was performed to ensure the validities of the parameters obtained in the LS-parallel test.

## 2. Theory

### 2.1. Advection-dispersion Model

In the advection-dispersion model, the transfer of a substance is represented by Equation (1) for a one-dimensional case:

(1) $$\begin{array}{c} \theta \frac{\partial C}{\partial t}=-\theta v\frac{\partial C}{\partial x}+\theta D\frac{\partial^{2}C}{\partial x^{2}}\;{-\rho }_{d}\frac{\partial M_{A}}{\partial t} \end{array}$$

where *θ* is the effective porosity (−), *C* is the concentration in the leachate (mg/L), *t* is the elapsed time (s), *v* is the actual flow velocity (m/s), *x* is a coordinate (m), *D* is the dispersion coefficient (m^2^/s), *ρ_d_* is the dry density (kg/L), and *M_A_* is the amount of the substance in question that is adsorbed on solids (mg/kg). *M_A_* is a function of *C*; its linear type (or Henry type) adsorption isotherm is shown in Equation (2):

(2) $$\begin{array}{c} M_{A}{=K}_{d}C \end{array}$$

where *K_d_* is the distribution coefficient (L/kg), which is peculiar to the contaminant in question [20]. Equation (3) is obtained from Equations (1) and (2):

(3) $$\begin{array}{c} \frac{\partial C}{\partial t}+\frac{v}{R}\frac{\partial C}{\partial x}=\frac{D}{R}\frac{\partial^{2}C}{\partial x^{2}} \end{array}$$

where *R* is the retardation factor (-), which is represented by Equation (4):

(4) $$\begin{array}{c} R=1+\frac{\rho_{d}}{\theta }K_{d} \end{array}$$

The analytical solution of Equation (3) in the case where clean water moves through a column filled with a PCM is:

(5) $$\begin{array}{c} C\left(x,t\right){=C}_{0}\left[1-\frac{1}{2}\left\{\mathrm{erfc}\left(\frac{\mathrm{Rx}-\mathrm{vt}}{2\sqrt{\mathrm{DRt}}}\right)+\exp\left(\frac{\mathrm{vx}}{D}\right)\mathrm{erfc}\left(\frac{\mathrm{Rx}+\mathrm{vt}}{2\sqrt{\mathrm{DRt}}}\right)\right\}\right] \end{array}$$

Equation (5) shows that the concentration at a focused point decreases with time from the initial concentration, *C*_0_. Some studies only describe the initial high concentration as “equilibrium” and describe the decrease curve as “non-equilibrium” (e.g., [17]), but this represents a misunderstanding. The monotonic decrease is always governed by the adsorption equilibrium represented by Equation (1) and including Equation (2), which is assuming an instant equilibrium. As a characteristic of the decrease curve, the larger the *K_d_*, the slower the decrease. *C*_0_ is calculated using Equation (6) [17]:

(6) $$\begin{array}{c} C_{0\;}=\frac{M_{T}}{K_{d}+\theta /\rho_{d}} \end{array}$$

where *M_T_* is the amount of a given substance taking part in the adsorption equilibrium (mg/kg).

In a previous study [17], it was assumed that all substances were present at the solid surface initially, and that they did not increase with time (e.g., through new dissolution at the solid surface). Thus, they represented the mass parameter in *C_s,ini_* (mg/kg), i.e., the solid-phase initial concentration. However, the present study expresses the parameter *M_T_* instead of *C_s,ini_* because *M_T_* often increases with time, which will be described further in Section 4.1.

### 2.2. Adsorption Isotherms of PCMs

Column percolation tests are considered necessary to obtain model parameters such as *M_T_* and *K_d_* [17]. The present study, however, aims to develop a procedure that only requires batch tests, which are simpler. The theory is as follows. The amount of a contaminant present in the liquid phase per mass of the solid, *M_L_* (mg/kg), can be expressed as follows:

(7) $$\begin{array}{c} M_{L\;}=\frac{V}{m}{\text{×}C=\mathrm{LS}}_{\mathrm{batch}}\text{×}C \end{array}$$

where *V* is the liquid volume (L), *m* is the mass of solid (kg), and *LS_batch_* is the ratio of liquid to solid in the batch condition (L/kg). Equation (7) shows that *M_L_* has a linear relationship with *C* with the proportional constant *LS_batch_*. The total amount of a substance per mass of solid taking part in adsorption equilibrium, *M_T_* (mg/kg), can be expressed as the sum of the substances adsorbed on the solid surface and those present in the liquid phase:

(8) $$\begin{array}{c} M_{T}{=M}_{A}{+M}_{L} \end{array}$$

Equation (9) can be obtained by substituting Equation (2) into Equation (8); when *K_d_* and *M_T_* are constant, *M_L_* can be represented as a function of *C*.

(9) $$\begin{array}{c} M_{L}{=-K}_{d}{C+M}_{T} \end{array}$$

In addition to a Henry type isotherm (Equation (2)), the relationship between *M_A_* (mg/kg) and *C* (mg/L) can also be represented by Freundlich type and Langmuir type isotherms, as shown in Equations (10) and (11), respectively [21]:

(10) $$\begin{array}{c} M_{A\;}{=K}_{F}C^{N} \end{array}$$

(11) $$\begin{array}{c} M_{A\;}=\frac{M_{\mathrm{sat}}K_{L}C}{{1+K}_{L}C} \end{array}$$

where *K_F_*, *N*, and *K_L_* represent constants (*N* < 1), and *M_sat_* represents the saturated adsorption amount (mg/kg). In all isotherms (Equations (2), (10), and (11)), the higher *C* (mg/L), the higher *M_A_* (mg/kg). Equations (12) and (13) can be obtained by substituting Equations (10) and (11), respectively into Equation (8):

(12) $$\begin{array}{c} M_{L}{=-K}_{F}C^{N}{+M}_{T} \end{array}$$

(13) $$\begin{array}{c} M_{L\;}=-\frac{M_{\mathrm{sat}}K_{L}C}{{1+K}_{L}C}\;{+M}_{T} \end{array}$$

**Figure 1:**
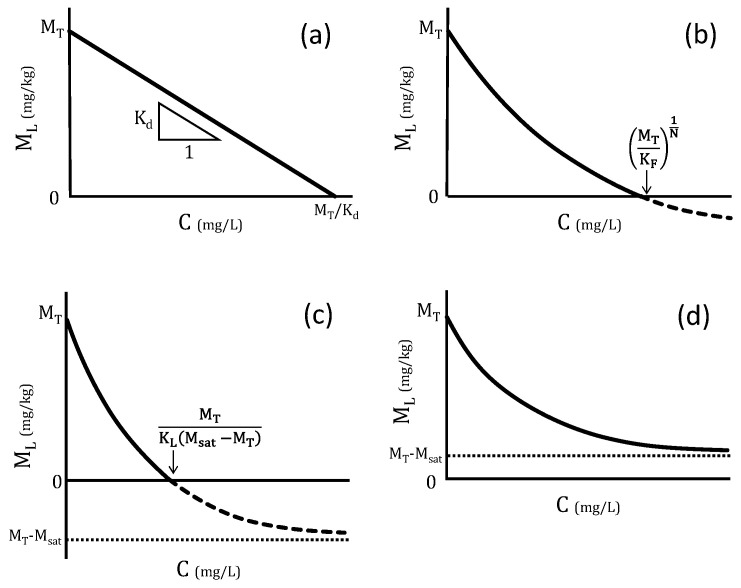
Schematic relationships between liquid phase concentration and eluted amount per mass in LS-parallel test. (**a**) Henry (linear) type, (**b**) Freundlich type, (**c**) Langmuir type of M_T_ < M_sat_, and (**d**) Langmuir type of M_T_ > M_sat_.

Figure 1 shows the relationship between *M_L_* and *C* in (a) Henry type, (b) Freundlich type, and (c) (d) Langmuir type isotherms. In panels (a–d), the Y-intercept represents *M_T_*. In panels (a–c), the X-intercept represents the liquid phase concentration, *C*, when the liquid volume, *V*, is zero (i.e., when the minimum amount of water is in contact with the dry material). In case of panel (d), there is no X-intercept as *M_T_* is larger than *M_sat_*, so *C* will rise until solubility is reached.

### 2.3. Application of LS-Parallel Test on PCMs

In a single batch test, one value for *C* is obtained, and *M_L_* can be calculated from Equation (7). Therefore, by performing several batch tests in parallel on one PCM mother sample, while changing the liquid-solid ratio (LS-parallel test), various *C* and *M_L_* values can be obtained. By plotting the relationship between *C* and *M_L_*, as shown in Figure 1, and by approximation of any of Equations (9), (12), or (13), the adsorption parameters of the PCM can be obtained.

This procedure was developed under the assumption that the mass of substance taking part in the adsorption equilibrium, *M_T_*, is constant. However, it should be noted that additional substances may be released from the solid phase over time and/or (co-)precipitated during a batch test. Another assumption is that the factors affecting adsorption equilibrium, such as pH, are the same among the batch tests conducted in parallel. The most important test condition would be duration, as these aspects might change dynamically with time. However, the adsorption isotherms (Equations (2), (10) and (11)) do not include time, indicating that the adsorption equilibrium is instantaneously achieved. Therefore, it is necessary to find the optimum LS-parallel test conditions, and especially the test time must be carefully examined.

## 3. Material and Methods

### 3.1. Material

A sandy soil derived from marine sediment in Japan, called a tsunami deposit, was used for analysis. This sample was obtained from a temporary stockpile from Miyagi prefecture after the 2011 East Japan Great Earthquake. As huge amounts (approximately 10 million tons) of tsunami deposits were generated after the disaster, it would be advantageous to recycle these tsunami deposits through civil engineering projects. However, the soil contains trace contaminants, and therefore evaluation of its environmental safety is necessary.

After sampling, the tsunami deposit was air-dried, sieved through a 2-mm sieve and stored at room temperature (25 °C) before the leaching tests. The particle density was 2.65 g/cm^3^, and the water content was 0.6%. Table 1 shows the elemental composition as determined by aqua regia extraction and alkali melting, followed by inductively coupled plasma optical emission spectrometry (ICP-OES; Model 720, Agilent Technologies Inc., Santa Clara, CA, USA) and inductively coupled plasma mass spectrometry (ICP-MS; 7500CX, Agilent Technologies Inc., Santa Clara, CA, USA) except for Si, P, S, and Cl, which were measured by fluorescent X-ray spectroscopy (Primus II, Rigaku Corp., Matsubara-cho Akishima, Japan).

### 3.2. LS-Parallel Test

The LS-parallel test comprises a series of batch test performed in parallel under different liquid-solid ratios. A LS-parallel test has been standardized in which the liquid-solid ratios are varied from 0.5 to 10 L/kg, the sample size is 2 mm or less, and the contact time is 48 h [22]. However, as shown in Table 2, here the test conditions were modified to accommodate wider ranges. The maximum liquid-solid ratio range was expanded to 300 L/kg, because the larger the liquid-solid ratio, the better the extrapolation prediction of *M_T_*. The range of contact time was also expanded widely, from 10 min to 28 d, to allow for analysis of changes in the eluate conditions. The test times used here, such as 10 min and 28 days, are not suitable for future standardization, but it is meaningful to explore such extreme conditions to consider the release mechanisms that occur during a batch test system. The mass of sample, volume of solvent, and volume of vessel for each test are shown in Table 2. For each test condition, two or three subsamples were applied. In total, 70 single batch tests were executed. The vessels were made from high-density polyethylene. The solvent used was a 1 mmol/L calcium chloride solution, which was applied to reduce the generation of colloids. Mixing was performed with a tumbling shaker at approximately 5 rev/min. After each test, the solution was immediately filtered using a polytetrafluoroethylene (PTFE) membrane filter with a pore size of 0.45 μm. To check the effect of colloids on the 0.45 μm-filtrate, a part of the filtrate was further filtered with a 0.1 µm PTFE membrane filter for a contact time of only 6 h.

### 3.3. Column Percolation Test

An acrylic column with an inner diameter of 5 cm was packed with 289 g of the soil sample, to obtain a thickness of 10 cm. This thickness deviates from the ISO 21268-3 up-flow percolation test, 30 ± 5 cm [3], due to the amount of the soil sample stored. As an eluent, a solution of 1 mmol/L calcium chloride was introduced from the bottom of the column using a peristaltic pump. A linear velocity of 15 ± 2 cm/d was applied, i.e., a flow rate of 288 ± 24 mL/d. Thus, an *LS_cum_* of approximately 1.0 L/kg of eluent passed through the column per day. The flow was continued after the eluent level reached top of the column, which marks another deviation from ISO 21268-3; in the standard, after filling the column with eluent, the apparatus should be left for two days to achieve “equilibrium”, and then the water flow should be restarted. In this study, non-stop flow was chosen to shorten the total test time; the LS-parallel test result justified that after two days the concentration was still changing, i.e., an equilibrium had not been reached. Under this non-equilibrium environment, the total time itself was important. Fourteen fractions of *LS_cum_* of approximately 0.1, 0.3, 0.4, 0.6, 0.9, 1.2, 1.5, 1.8, 2.5, 3.1, 3.7, 5.2, 6.8, and 8.3 L/kg were taken to elucidate the release behavior of substances from the soil sample. The collected eluates were filtered using PTFE membrane filter with a pore size of 0.45 µm.

### 3.4. Measurement

The pH and electrical conductivity (EC) of the filtrate were measured immediately. Cl^−^, F^−^, PO_4_^3−^, and SO_4_^2−^ were determined by ion chromatography (Dionex ICS 2100, Thermo Fisher Scientific Inc., Waltham, MA, USA). Al, As, B, Ba, Ca, Co, Cr, Cu, Fe, K, Mg, Mn, Mo, Na, Ni, Pb, Rb, Sb, Se, Si, Sr, Ti, and Zn were determined by ICP-OES (Model 720, Agilent Technologies Inc.) or ICP-MS (7500CX, Agilent Technologies Inc.), depending on their concentration level.

## 4. Results and Discussion

### 4.1. Changes in pH with Time during the LS-Parallel Test

**Figure 2:**
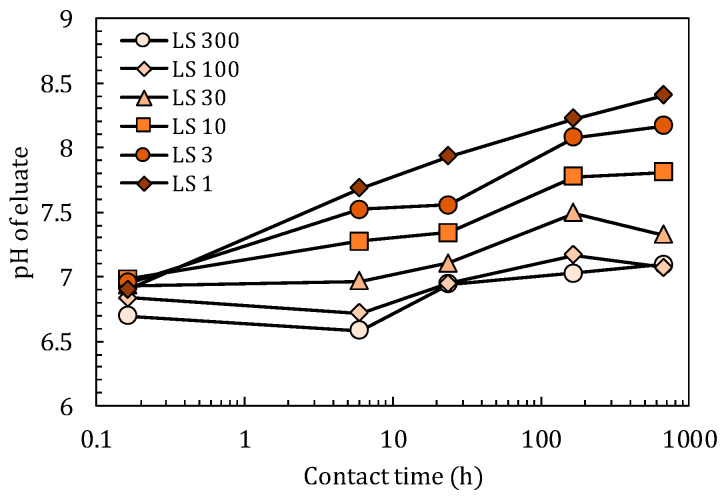
Change in pH in the LS-parallel test as a function of contact time.

As pH significantly affects equilibria, such as the precipitation-dissolution and adsorption–desorption equilibria, it is desirable to restrict pH changes to a narrow range as possible among all eluates. This helps to obtain one parameter from a set of batch tests with differing conditions, such as liquid-solid ratio and contact time. Figure 2 shows the changes in pH with time during the LS-parallel test. Up until just after 10 min, pH remained in a narrow range (6.7–7.0). After this point, however, pH increased with time; the trends were clearer under lower *LS* conditions (hereinafter *LS* means *LS_batch_* if there is no other notification). In *LS* 1, for example, pH reached 8.4 after 28 days. Larger *LS* conditions, such as 100 and 300 L/kg, showed relatively constant pH at approximately 7.0.

It may be possible to control pH values by adding acid or alkali solutions. However, pH was not controlled in this study, because the concentrations of acid or alkali and the frequency of addition must be carefully adjusted to reduce fluctuations in pH. Furthermore, until the acid or alkali solution was sufficiently diluted in the mixture, high concentrations of the solution may have directly affected soil particles. Such results were experienced in preliminary batch tests. Therefore, in the results hereinafter it is necessary to consider that pH exhibited a range of approximately 1.8 during the batch test, which might have affected the equilibria of substances.

### 4.2. Approximation of Adsorption Parameters from LS-Parallel Test

**Figure 3:**
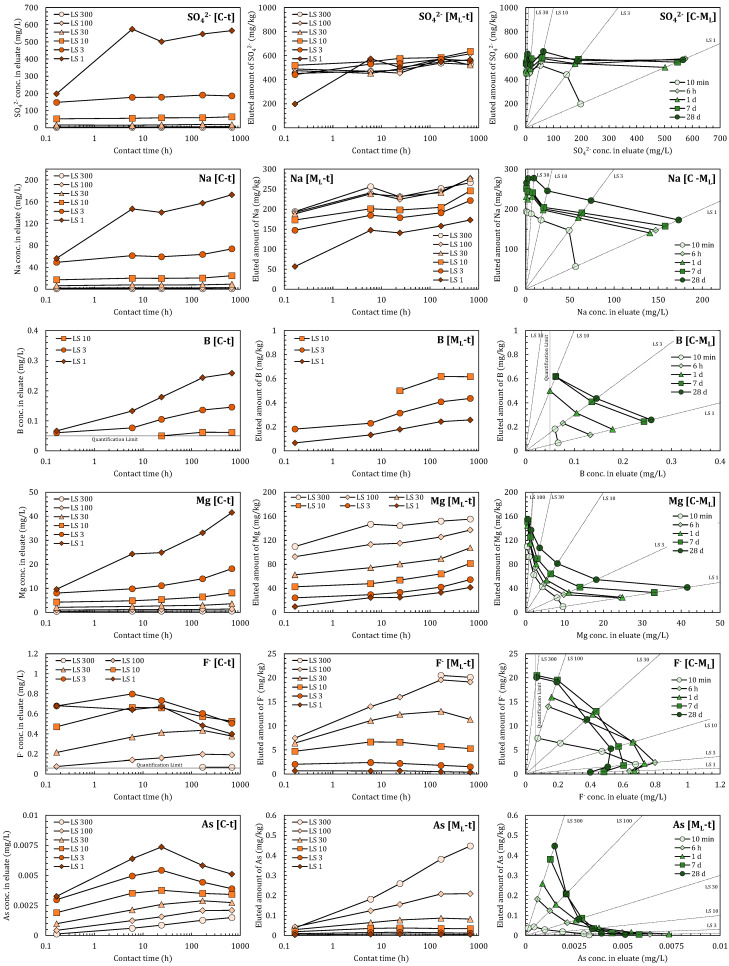
Changes in eluate concentration with time [*C-t*], changes in eluted amount with time [*M_L_-t*], and relationship between eluate concentration and eluted amount [*C-M_L_*]. In [*C-M_L_*] panels, data of the same *LS_batch_* condition are plotted on a straight-line passing through the origin. A lack of data means the eluate concentration was below the quantification limit.

Figure 3 shows the mutual relationships [*C-t*], [*M_L_-t*], and [*C-M_L_*], as obtained by LS-parallel tests on SO_4_^2−^, Na, B, Mg, F^−^, and As. Regarding the relationship [*C-t*], the smaller the *LS*, the higher the eluate concentration, *C*. In Japan’s soil environmental standard, B, F^−^, and As are designated as regulated substances. They are judged by a batch test mixing with pure water with a liquid-solid ratio of 10 L/kg for 6 h. The reference values of B, F, and as are 1.0, 0.8, and 0.01 mg/L, respectively [23]. 

The [*M_L_*-*t*] relationships were determined by converting *C* to *M_L_* using Equation (9), as shown in the panels in the center column of Figure 3. Generally, larger *LS* values corresponded to higher *M_L_*, with the exceptions of SO_4_^2−^ and Na. The results coincided for SO_4_^2−^ except for at 10 min in *LS* 1, whereas for Na they coincided above *LS* 30. The results obtained after 10 min in *LS* 1 appeared extremely low, presumably because even soluble substances could not be dissolved sufficiently at this time.

Using the results of *C* and *M_L_*, the [*C-M_L_*] relationships were plotted as shown in the left-hand column of Figure 3. SO_4_^2−^ showed almost horizontal, linear relationships. Referring to Figure 1, the slope representing *K_d_* was almost zero, indicating that adsorption did not work for SO_4_^2−^. The parameters approximated from the [*C-M_L_*] relationships are summarized in Table 2. For SO_4_^2−^, *K_d_* was almost zero and *M_T_* remained almost unchanged until 28 days.

For Na, B, and Mg, [*C-t*] and [*M_L_-t*] showed monotonous increases among all *LS* conditions (for B in *LS* 30, 100, and 300 all eluates were below the quantification limit). Such increases imply additional releases from the solid to the elution. The [*C*-*M_L_*] relationships of Na and B appeared to be linear in each *LS* data set, indicating that they exhibited Henry type adsorption isotherms. Mg exhibited a curved relationship that fitted well with a Langmuir type isotherm. All relationships gradually increased with time, indicating that the *M_T_* of each substance increased gradually. The parameters obtained by fitting are summarized in Table 3. The observed gradual increases might represent intraparticle diffusion from inside the solid to the surface [24,25]. This could have proceeded with time due to differences in concentration inside and outside of the solid [26]. Besides, the adsorption isotherms shown in Equations (2), (10), and (11) feature no time-parameters, meaning that adsorption was considered to be in an instant equilibrium. Such differences in release mechanisms will be discussed in Section 4.4.

In case of F^−^ and As, [*C-t*] and [*M_L_-t*] initially increased, but after a certain period then began to decrease. Such behaviors were also observed for Co, Cu, Mo, Ni, Sb, and Se. Lower *LS* conditions (higher *C* conditions) appeared to result in the observed decrease occurring earlier, which might have been due to precipitation and/or coprecipitation in batch test conditions [27]. Changes in the pH of the eluate (Figure 2) could also have affected the behaviors of pH-sensitive substances. SO_4_^2−^, Na, Mg, and B would not be so sensitive to precipitation, coprecipitation, or adsorption around the pH ranges observed in Figure 2. To confirm this assumption, however, a successful pH-adjusted batch test would be necessary. As a result, most parts of the [*C-M_L_*] relationships of F^−^ and As appeared irregular due to concentration decreases. Therefore, such ranges should be removed when estimating adsorption parameters. In Table 3, the parameters of F^−^ and As were approximated using the data obtained before the observed decreases in concentration.

LS-parallel tests should be conducted under moderately short contact time (neither too short, such as 10 min, nor too long such as, >7 d) to obtain adsorption parameters. A contact time between 6 h and 1 d seemed to deliver the best results. Higher liquid-solid ratios could better maintain adsorption control and small changes in pH, although the concentration in the eluate could be lower than the quantification limit.

Although there are few studies on adsorption parameters for PCMs, the *K_d_* of soluble salts such as Na, K, SO_4_^2−^, and Cl^−^ are estimated to be small, as in Table 3; for contaminated soils, the *K_d_* of SO_4_^2−^, Cl^−^, Cu, and ΣPAH are calculated to be 0.55, 0.50, 0.60, and 120, respectively, by fitting the advection-dispersion model of column test data [17]. Similarly, for APC residues, the *K_d_* of Na, K, and Cl^−^ were calculated to be 1.3, 0.83–1.3, and 0.55 from column test data [7]. In addition, Reference [7] performed an LS-parallel test in the *LS_batch_* range of 5–500 L/kg and calculated the *K_d_* of Na, K, and Cl^−^ as 0.20–1.0, 0.29–1.3, and 0.38–6.0, respectively, by fitting the advection-dispersion model as a function of *LS_cum_*. However, the handling of physical parameters such as dispersion length and porosity is unclear, so calculations using the procedure proposed in this study are expected.

### 4.3. Effect of Colloids Passing the Filter

Colloids could have passed through the filter during the liquid-solid separation step, and so may have affected the measured concentrations in the eluate [28,29,30]. To confirm the effect of colloids, eluates obtained using a 0.45 μm pore size filter (0.45 MF) were refiltered using a 0.1 μm pore size filter. This procedure was applied to eluates using only a 6 h-mixing time.

**Figure 4:**
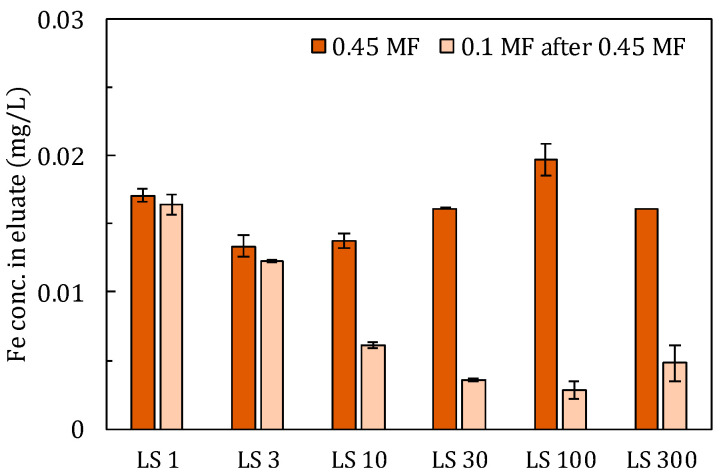
Concentration of Fe in filtrate using 0.45 µm membrane filter (MF) and in filtrate after re-filtration of 0.45 µm filtrate using the 0.1 µm membrane.

As shown in Figure 4, in *LS* 1 and 3, refiltration did not affect Fe concentration significantly, but under larger *LS* conditions concentrations clearly decreased in following refiltration. This suggests that in smaller *LS* conditions, colloids of 0.45 μm or smaller were removed at the first filtration by the cake that formed on 0.45 MF, but in larger *LS* conditions, such cake did not foam sufficiently to remove the colloids. Subsequent refiltration using 0.1 MF was able to remove colloids of 0.1–0.45 mm, resulting in significant reductions of their concentrations in the eluates [28]. Furthermore, in *LS* 300 it may have been difficult to remove colloids of 0.1 μm or smaller during refiltration, as the resulting concentration was higher and the observed variation was larger than those of *LS* 100. Ti and Al showed similar trends, and Pb and Zn also fluctuated significantly; these substances should therefore be excluded from the evaluation of adsorption parameters.

Care should be taken to properly remove colloids during the solid–liquid separation step, for example, by applying centrifugation with sufficient intensity and duration. Furthermore, considering the real environment, it would be necessary to develop a solid–liquid mixing method that minimizes the further generation of colloids through the abrasion of solids.

### 4.4. Reproductivity of Column Percolation Test by LS-Parallel Test

**Figure 5:**
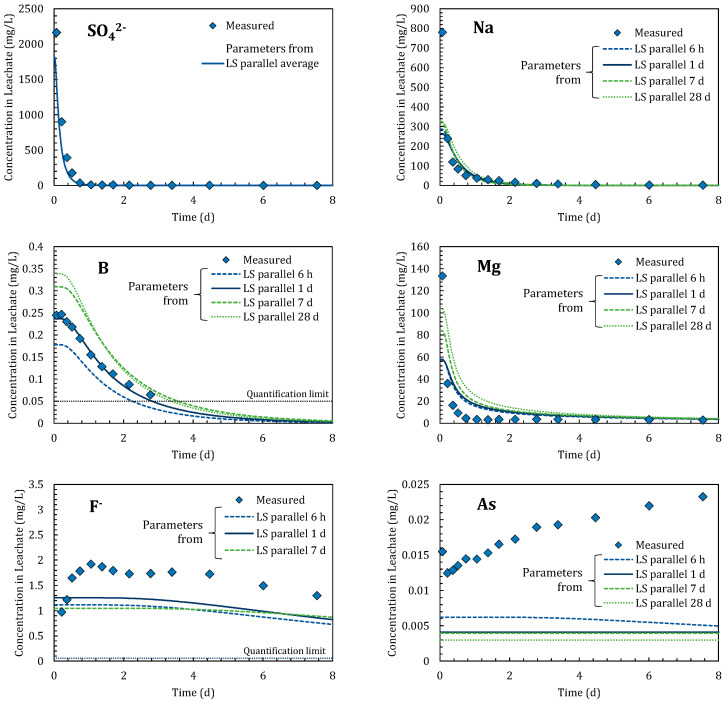
Column percolation test results and calculation results using parameters obtained in the LS-parallel tests.

Figure 5 shows the changes in eluate concentration in the column percolation test, together with the results calculated using parameters from the LS-parallel test. In these calculations, the effective porosity, *θ*, and dispersion length, *α*, were estimated as 0.286 (−) and 0.0387 m, respectively, from the fitting of SO_4_^2−^. This was because *K_d_* was almost zero and *M_T_* did not increase over time during the LS-parallel test. In Figure 5, all parameters shown in Table 2 were used for calculations, because *K_d_* and *M_T_* certainly changed over time during not only the batch test, but also during column percolation tests (except for SO_4_^2−^). Therefore, the horizontal axis in Figure 5 shows the elapsed time. It should be noted that 1 day is almost equivalent to 1 *LS_cum_*, as the sample volume of the column was 289 g and the water flow rate was 288 mL/d.

The maximum concentration is one of the most important parameters in evaluating the environmental impact of a given substance [31]. In the advection-dispersion model, the eluate concentration decreased monotonically. Thus, the first eluate represented the maximum concentration, which can be calculated using Equation (6). In Figure 5, SO_4_^2−^, Na, B, and Mg showed their maximum concentrations in the first or the second eluate fractions. The calculated concentrations obtained by parameters from the LS-parallel test after one day were 67%, 38%, 96%, and 44% of measured SO_4_^2−^, Na, B, and Mg values in the column test, respectively; this timing would provide the best comparison to the measured results because the initial run of the eluate took 16.3 h. For SO_4_^2−^, Na, and B the decrease curves showed good agreement with the measured values. Ba, Co, Cr, Cu, K, Rb, Ni, Se, Si, Sr, and Zn also showed typical monotonic decreases. However, in the LS-parallel test, the concentrations of Co, Cr, Cu, Rb, Ni, Se, and Zn significantly fluctuated with time, probably due to the effects of colloids. In the column test, colloids originally contained in the soil would have been unlikely to spill out due to self-clogging. Furthermore, soil particles were not eroded during percolation. These results suggest that if the water-mixing procedure in the LS-parallel test were to be improved, the adsorption parameters of these substances could be obtained.

The eluate concentration of F^−^ increased from 0.97 to 1.9 mg/L, and then decreased gradually. Al, Mo, Sb, and Ti also showed peaks during the midpoint of their runs, and then decreased. The As concentration continued to rise until the end of the column test period. Fe and Mn also continued to rise from the first eluate to the end. However, the current advection-dispersion model could not simulate such partially or totally increasing trends because it only assumes an adsorption–desorption equilibrium. As seen in the LS-parallel tests conducted with different water-contact times, in the column test it could also be expected that dissolution and/or intraparticle diffusion was occurring. The advection-dispersion model should thus be developed to further consider these mechanisms.

### 4.5. Further Applications of LS-Parallel Test

The theory of obtaining the adsorption–desorption parameters of PCMs through the LS-parallel test is quite clear. Comparing the LS-parallel test and column test results confirmed that this theory is applicable to substances in which the adsorption–desorption equilibrium is dominant (SO_4_^2−^, Na, Mg, etc.). It is presumed that precipitation hardly occurred for these substances, and that the adsorption–desorption equilibrium was not significantly affected in the observed pH range.

The advantages of the proposed method are: (1) the parallel batch test is simpler and easier to conduct than the column percolation test, which means that the LS-parallel test could replace column percolation test to obtain the parameters; (2) as contaminants are released from the material itself, further addition of the contaminants is unnecessary, and the real chemical species from the PCM can be considered; and (3) the advection–related parameters are obtained at once for every substance released from the PCM, assuming the substance exhibits precipitation-free and adsorptive characters.

Regarding its further applications, the LS-parallel test can easily evaluate changes in the adsorption parameters of PCMs through specific conditions. For example, the mechanism could be investigated by LS-parallel test whether *M_T_* decreases or *K_d_* increases when a contaminant in a PCM is insolubilized with chemicals. The long-term stability of substances under weathering conditions could also be evaluated by analyzing a fewer amount of PCMs than would be required if using column percolation tests.

## 5. Conclusions

This study proposed a technique to determine the adsorption parameters using an LS-parallel test for materials that release contaminants. LS-parallel and column percolation tests were performed on a sandy soil derived from marine sediment.

In the LS-parallel test, adsorption parameters were successfully obtained if the substance was under adsorption control. Combining batch test conditions with the liquid-solid ratio and water contact time permits the investigation of leaching mechanisms from the inside of the solid phase, and of precipitation or coprecipitation reactions in the liquid phase after leaching. In conclusion, LS-parallel tests should be conducted under moderately short contact time; between 6 h and 1 d seemed to deliver the best results. Higher liquid-solid ratios could better maintain adsorption control and small changes in pH, although the concentration in the eluate could be lower than the quantification limit.

In the column percolation test, the behavior of SO_4_^2−^ and B coincided well with the advection-dispersion model using adsorption parameters obtained from the LS-parallel test. However, for other substances, the initial concentration and release curves did not always fit, probably because these substances were continuously released from the soil, or because colloids affected differently between the LS-parallel tests and the column percolation test. These results suggest that improvements are necessary in the mixing method of the LS-parallel test procedure to suppress the release of colloids from the solids. Additionally, the advection-dispersion model should further be improved to express the release of substances from inside the solids.

As observed in the experiments, the adsorption parameters can change with time under different exposure conditions. Since the LS-parallel test is easy to apply, the combination of the LS-parallel test and the analysis method proposed in this study can be a very powerful tool to evaluate the changes in adsorption parameters and, moreover, the impact of PCMs on the environment.

## Figures and Tables

**Table 1:** Elemental composition of sandy soil.

	Content			Method		Content (mg/kg)			Method
	(% for Si-P, mg/kg for S-Sr)								
Si	24.1			XRF	Zn	98.7	±	2.9	AD + AF
Al	7.20	±	0.26	AD + AF	Rb	66.0	±	1.6	AD + AF
Fe	3.72	±	0.09	AD + AF	Cr	52.9	±	8.6	AD + AF
K	3.19	±	0.18	AD + AF	Cu	25.6	±	0.5	AD + AF
Na	2.25	±	0.19	AD + AF	Ni	18.3	±	0.6	AD + AF
Ca	2.12	±	0.12	AD + AF	Pb	16.5	±	0.2	AD + AF
Mg	1.02	±	0.020	AD + AF	Se	14.8	±	0.5	AD + AF
Ti	0.331	±	0.023	AD + AF	As	12.7	±	1.3	AD + AF
P	0.120			XRF	Co	12.5	±	0.3	AD + AF
S	884			XRF	Cs	3.15	±	0.48	AD + AF
Mn	762	±	32	AD + AF	Mo	0.96	±	0.03	AD + AF
Cl	540			XRF	Sb	0.35	±	0.01	AD + AF
Ba	525	±	13	AD + AF	Cd	0.20	±	0.00	AD + AF
Sr	313	±	18	AD + AF					

AD + AF: acid digestion and alkali fusion.

**Table 2:** Summary of LS-parallel test conditions.

	Liquid-Solid Ratio (L/kg)	Sample Amount (g)	Solution Volume (mL)	Vessel Volume (mL)	Replicates	Contact Time
LS 1	1	60	60	250	2	10 min, 6 h, 1 d, 7 d, 28 d
LS 3	3	20	60	250	2	10 min, 6 h, 1 d, 7 d, 28 d
LS 10	10	10	100	250	2	10 min, 6 h, 1 d, 7 d, 28 d
LS 30	30	5	150	250	2	10 min, 6 h, 1 d, 7 d, 28 d
LS 100	100	5	500	1000	3	10 min, 6 h, 1 d, 7 d, 28 d
LS 300	300	2.5	750	1000	3	10 min, 6 h, 1 d, 7 d, 28 d

**Table 3:** Parameters obtained from LS-parallel tests.

	Duration Time in LS-Parallel Test	Referred LS Range	Referred Concentration Range	M_T_	Henry	Langmuir		Coefficient of Determination R^2^
					K_d_	M_sat_	K_L_	
		(L/kg)	(mg/L)	(mg/kg)	(L/kg)	(mg/kg)	(L/mg)	
SO_4_^2−^	6 h	1–10	50–600	530	0.06	-	-	0.604
	1 d	1–10	60–500	580	0.15	-	-	0.884
	7 d	1–300	2–500	570	0.03	-	-	0.153
	28 d	1–300	2–600	570	0.01	-	-	0.001
Na	10 min	3–300	0.6–50	190	0.96	-	-	0.988
	6 h	1–10	20–150	210	0.42	-	-	0.999
	1 d	1–10	20–140	210	0.47	-	-	1.000
	7 d	1–10	20–160	210	0.34	-	-	1.000
	28 d	1–10	25–170	260	0.49	-	-	1.000
B	6 h	1–3	0.08–0.1	0.36	1.7	-	-	-
	1 d	1–3	0.1–0.2	0.51	1.8	-	-	-
	7 d	1–10	0.06–0.2	0.72	2.0	-	-	0.971
	28 d	1–10	0.06–0.3	0.72	1.8	-	-	0.993
Mg	10 min	3–300	0.4–8.0	120	-	140	0.34	0.996
	6 h	1–100	1.1–25	170	-	160	0.65	0.994
	1 d	1–100	1.2–25	170	-	160	0.50	0.999
	7 d	1–100	1.3–33	170	-	150	0.37	0.995
	28 d	1–100	1.4–41	160	-	140	0.18	0.999
F^−^	10 min	10–100	0.07–0.5	7.9	6.9	-	-	0.999
	6 h	30–100	0.1–0.4	16	14	-	-	-
	1 d	30–100	0.2–0.4	18	14	-	-	-
	7 d	30–300	0.07–0.4	23	21	-	-	0.947
As	10 min	1–300	0.0001–0.003	0.043	13	-	-	0.961
	6 h	1–300	0.0006–0.006	0.25	-	0.3	480	0.995
	1 d	30–300	0.0009–0.003	0.47	-	0.7	520	0.997
	7 d	30–300	0.001–0.003	1.3	-	1.6	980	0.932
	28 d	100–300	0.001–0.002	2.9	-	3.6	1500	-

## Data Availability

Data sharing is not applicable to this article.
